# Correction to: Human ovarian cancer intrinsic mechanisms regulate lymphocyte activation in response to immune checkpoint blockade

**DOI:** 10.1007/s00262-020-02601-z

**Published:** 2020-06-18

**Authors:** Marina Natoli, Nair Bonito, James D. Robinson, Sadaf Ghaem-Maghami, Yumeng Mao

**Affiliations:** 1 Department of Surgery and Cancer, Institute of Reproductive and Developmental Biology, Imperial College London, London, UK; 2 Mechanistic Biology and Profiling, Discovery Sciences, R&D, AstraZeneca, Cambridge, UK; 3 Bioscience, Early Oncology R&D, AstraZeneca, Cambridge, UK; 4 Science for Life Laboratory, Department of Immunology, Genetics and Pathology, Uppsala University, Uppsala, Sweden

## Correction to: Cancer Immunology, Immunotherapy https://doi.org/10.1007/s00262-020-02544-5

The original version of this article unfortunately contained a mistake. Complete figure captions are missing. Figures 1, 2, 3, 4 and 5 with complete figure caption are placed in the following pages:

**Fig. 1:**
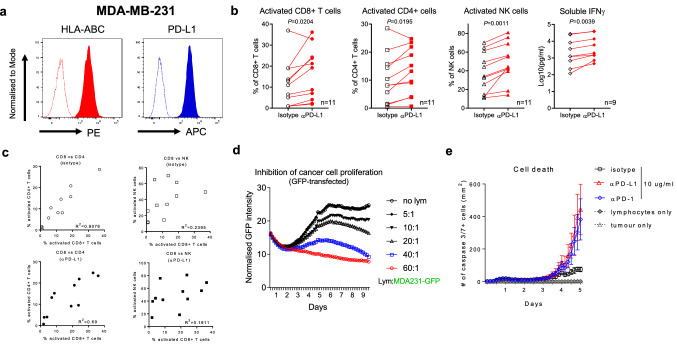
Immune checkpoint blockade enhances tumour cell killing mediated by primary human lymphocytes. **a** Surface expression of HLA-ABC and PD-L1 on human breast cancer line MDA-MB-231 is determined by FACS. **b** Primary human lymphocytes from 11 donors are co-cultured with MDA-MB-231 cells ± αPD-L1 mAb. The resulted activation of CD8 + T cells, CD4 + T cells and NK cells is measured by FACS, and the levels of soluble IFNγ are measured by ELISA on day 6. **c** Correlations between activation of CD8 + T cells and CD4 + T cells or CD8 + T cells and NK cells are analysed with or without αPD-L1 mAb. **d** GFP-transfected MDA-MB-231 cells are cultured alone or co-cultured with increasing numbers of primary human lymphocytes, and growth inhibition is quantified in real time by IncuCyte. **e** MDA-MB-231 and primary human lymphocytes are cultured alone or in TICS with the addition of isotype control antibody, αPD-L1 or αPD-1 mAb, and the resulted cell death is quantified in real time using a caspase 3/7 green dye in the IncuCyte. Data are presented as mean ± SD based on 3 technical replicates. Statistical differences are analysed using paired *T* tests

**Fig. 2:**
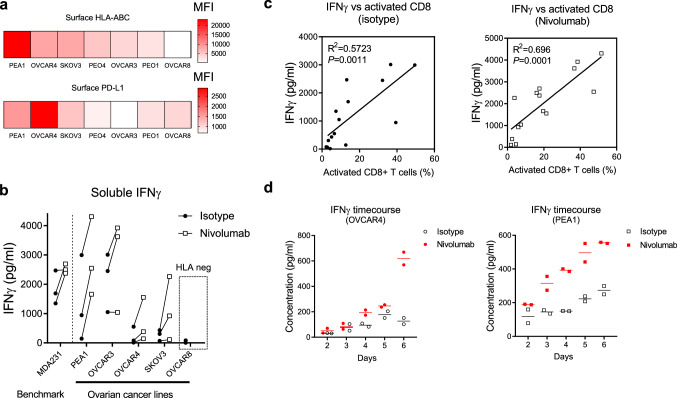
Human ovarian cancer cell lines are responsive to nivolumab in TICS. **a** Surface expression of HLA-ABC or PD-L1 is measured on a range of human ovarian cancer cell lines and the average mean fluorescence intensity (MFI) values from 3 independent experiments are presented. **b** Selected human ovarian cancer cell lines are co-cultured with primary human lymphocytes from 3 donors with isotype control antibody or nivolumab (10 μg/ml). MDA-MB-231 cells were also included as a positive control. Soluble levels of interferon-γ (IFNγ) are measured using ELISA on day 6. **c** Correlation between the percentages of activated CD8 + T cells and levels of soluble IFNγ are analysed in presence of isotype control or nivolumab. **d** Human ovarian cancer cell lines OVCAR4 or PEA1 are co-cultured with primary human lymphocytes in presence of isotype control antibody or nivolumab (10 μg/ml) in duplicate wells. Supernatants are collected and the levels of soluble IFNγ are determined by ELISA on day 2, 3, 4, 5 and 6

**Fig. 3:**
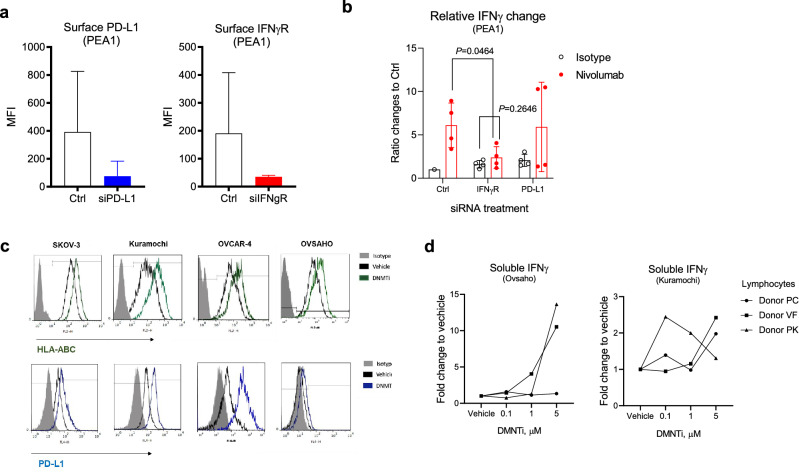
HLA and PD-L1 regulate cancer-driven lymphocyte activation. **a** siRNA targeting human PD-L1 and IFNγ receptor decrease surface protein expression (n = 4, mean ± SD). **b** Treatment naïve PEA1 cancer cells are first treated with siRNAs and then tested in TICS with an isotype control antibody or nivolumab (10 μg/ml). The resulted levels of soluble IFNγ are measured on day 6 in 4 independent experiments (mean ± SD). **c** Selected human OC cell lines are treated with vehicle or a DNMT inhibitor (5 μM) and the expression of surface HLA-ABC and PD-L1 are measured by FACS. **d** Human OC cancer cell lines, Ovasho and Kuramochi, are treated with vehicle or indicated concentrations of DNMT inhibitor. On day 8, the cells are harvested and washed then tested in TICS with 3 primary donor lymphocytes. Levels of soluble IFNγ are determined on day 6. Each data point is shown as mean from two duplicate wells and as fold change from vehicle control. Statistical differences are analysed using paired *T* tests

**Fig. 4:**
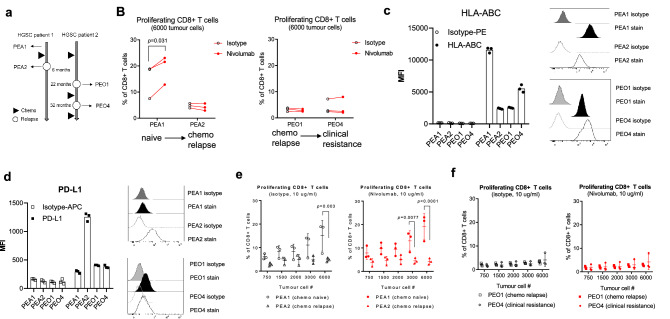
Reduced HLA expression in platinum resistant human OC cells limits PD-1 blockade. **a** Illustration of the generation of paired OC cell lines. **b** Activation of CD8 + T cells in TICS using the paired OC cell lines in presence of an isotype control antibody or nivolumab (10 μg/ml). Surface expression of **c** HLA-ABC and **d** PD-L1 is determined using FACS in 3 independent experiments. Representative histograms are also shown. Various lymphocyte-to-cancer cell ratios are tested in TICS using the **e** PEA-1/2 pair and **f** the PEO1/4 pair in presence of an isotype control antibody or nivolumab (10 μg/ml). Statistical analyses are performed using paired *T* test

**Fig. 5:**
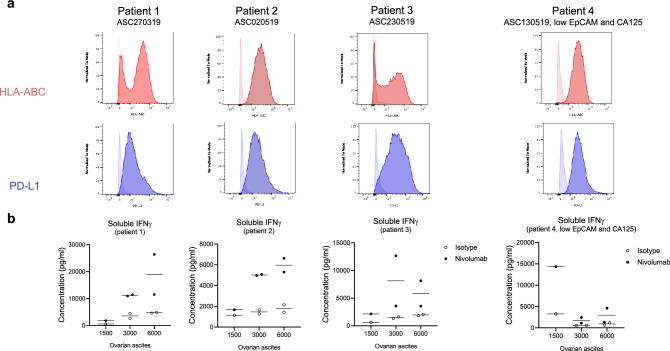
Treatment naïve primary OC cells respond to PD-1 blockade in TICS. **a** Surface expression of HLA-ABC and PD-L1 on 4 purified human OC ascites samples. **b** Primary OC cells are co-cultured with primary human lymphocytes in presence of an isotype control antibody or nivolumab (10 μg/ml) at different ratios in duplicate wells. The resulted soluble IFNγ levels are determined on day 6 by ELISA

